# HDL-related lipid ratios reflecting metabolic inflammation are associated with endometriosis status and ASRM stage

**DOI:** 10.3389/fphys.2026.1723135

**Published:** 2026-02-20

**Authors:** Qian Yang, Ming Ai, Huan Yang, Bixia Chen, Jinfa Huang, Kaixian Deng

**Affiliations:** Department of Gynecology, The Eighth Affiliated Hospital, Southern Medical University (The First People’s Hospital of Shunde, Foshan), Foshan, China

**Keywords:** ASRM stage, endometriosis, LHR, MHR, NHHR, NHR

## Abstract

**Background:**

Inflammation is central to the pathogenesis of endometriosis. High-density lipoprotein (HDL)-related lipid ratios have been proposed as indicators of systemic metabolic inflammation, yet their associations with endometriosis status and ASRM stage remain unclear.

**Methods:**

A total of 5,161 women were included, comprising 113 surgically confirmed endometriosis patients and 5,048 women without endometriosis. Multivariable logistic regression was used to evaluate the associations between four HDL-related lipid ratios—lymphocyte-to-HDL ratio (LHR), monocyte-to-HDL ratio (MHR), neutrophil-to-HDL ratio (NHR), and non-HDL-to-HDL ratio (NHHR)—and endometriosis status, with propensity score matching (PSM) applied to assess robustness. Among women with endometriosis, analyses were restricted to 105 patients with complete revised American Society for Reproductive Medicine (ASRM) staging data. Multivariable logistic regression was used to examine the associations between HDL-related lipid ratios and ASRM stage (early vs. advanced), with inverse probability of treatment weighting (IPTW) applied for robustness assessment. Receiver operating characteristic (ROC) curves were constructed to evaluate discriminative performance.

**Results:**

LHR was inversely associated with endometriosis status (OR = 0.52, 95% CI: 0.30–0.90), whereas MHR (OR = 3.30, 95% CI: 2.02–5.39), NHR (OR = 4.32, 95% CI: 2.77–6.78), and NHHR (OR = 2.14, 95% CI: 1.20–3.78) were positively associated. All four HDL-related lipid ratios were significantly associated with advanced ASRM stage relative to early-stage disease, including LHR (OR = 3.97, 95% CI: 1.14–15.7), MHR (OR = 6.60, 95% CI: 1.96–26.8), NHR (OR = 4.65, 95% CI: 1.50–16.8), and NHHR (OR = 8.03, 95% CI: 1.47–54.3). PSM and IPTW confirmed the robustness of these associations. ROC results showed that MHR had the highest AUC for ASRM stage classification (0.763), whereas NHR had the highest AUC for endometriosis status classification (0.715).

**Conclusion:**

HDL-related lipid ratios are significantly associated with endometriosis status and ASRM stage. These findings suggest the potential utility of HDL-related lipid ratios as adjunctive indicators for clinical stratification.

## 1 Introduction

Endometriosis is an estrogen-dependent chronic inflammatory disease characterized by the ectopic implantation of endometrial-like tissue outside the uterine cavity. Clinically, it often presents as chronic pelvic pain, dysmenorrhea, infertility, and reduced quality of life (Allaire et al., 2023; As-Sanie et al., 2025). The condition affects up to 10% of women of reproductive age, with a prevalence as high as 30%–50% among infertile women (Kvaskoff et al., 2015; Van Gestel et al., 2024). Although endometriosis is not life-threatening, its high recurrence rate, delayed diagnosis, and limited treatment options make it a major gynecological public health concern worldwide (Van Gestel et al., 2024; Leone Roberti Maggiore et al., 2024). Currently, clinical diagnosis relies primarily on laparoscopy, and there is a lack of simple, reliable biomarkers for early screening and disease stratification (Saunders and Horne, 2025; Vallvé-Juanico et al., 2022; Terry et al., 2025).

Emerging evidence suggests that dysregulated lipid metabolism may play a role in the development and progression of endometriosis (Zolbin et al., 2019; Chen et al., 2025; Ma et al., 2025). High-density lipoprotein (HDL) is not only central to reverse cholesterol transport but also possesses antioxidant, anti-inflammatory, and immunomodulatory properties, contributing to vascular homeostasis and tissue protection (Higashi, 2023; Erdoğan et al., 2023). Previous studies have indicated that HDL cholesterol (HDL-C) may exert a protective effect against endometriosis (Melo et al., 2009; Wang et al., 2024; Saei Ghare Naz et al., 2024; Peng et al., 2024). However, under conditions of chronic inflammation or metabolic stress, HDL particles may undergo structural and functional remodeling, shifting from a protective to a pro-inflammatory phenotype and thereby perpetuating local inflammation (Chiesa et al., 2019; Yu et al., 2019; Rysz et al., 2020). Based on this mechanism, several composite HDL-related lipid ratios have been proposed—such as the lymphocyte-to-HDL ratio (LHR), monocyte-to-HDL ratio (MHR), neutrophil-to-HDL ratio (NHR), and non-HDL-to-HDL ratio (NHHR)—which are thought to simultaneously reflect lipid burden and systemic inflammation. These indices have shown promising predictive value in various cardiovascular, metabolic, and immune-related disorders (Zhao et al., 2024; Sun et al., 2024; Li et al., 2025; Xiong and Yu, 2025; Liang et al., 2025), suggesting that they may also be associated with endometriosis status at a systemic inflammatory level.

Given the inflammatory nature of endometriosis, it is reasonable to hypothesize that these HDL-related lipid ratios may not only be associated with endometriosis status but also with disease stage. The revised staging system proposed by the American Society for Reproductive Medicine (ASRM) is widely used to assess lesion extent and is commonly regarded as a clinical classification system reflecting the anatomical distribution of endometriotic lesions. However, the relationship between HDL-related lipid ratios and ASRM staging remains underexplored, with limited systematic investigation or definitive evidence.

To address this gap, the present study focused on patients with endometriosis confirmed by laparoscopy or surgical pathology. We aimed to investigate the associations between four HDL-related lipid ratios (LHR, MHR, NHR, and NHHR) and endometriosis status as well as ASRM stage. Our objective was to evaluate whether these inflammation-related lipid indices could serve as potential adjunctive biomarkers for clinical stratification, thereby offering additional insights into disease management.

## 2 Methods

### 2.1 Study design and population

This study was designed as a single-center, retrospective case–control study. The case group (n = 113) consisted of women diagnosed with endometriosis who received treatment at The Eighth Affiliated Hospital of Southern Medical University between 2020 and 2025. Eligible patients were aged 20–50 years and had endometriosis confirmed by laparoscopic findings and/or postoperative histopathological examination. Exclusion criteria included a history of malignancy, autoimmune diseases, acute inflammatory conditions, or other severe systemic diseases that could substantially affect metabolic status. In addition, participants with a current or prior history of smoking or alcohol consumption were excluded prior to analysis. The control group (n = 5,048; Table 1) was retrospectively recruited during the same period from women undergoing routine health examinations at the hospital’s Health Screening Center. Inclusion criteria for controls were an age range of 20–50 years, absence of gynecological symptoms in medical records, and no abnormal findings suggestive of potential endometriosis on pelvic imaging or gynecological examination. The exclusion criteria were identical to those applied to the case group.

**TABLE 1:** Baseline characteristics of study participants according to endometriosis status.

Variables	All n = 5,161	Control n = 5,048	Endometriosis n = 113	P value
LHR	1.42 (0.49)	1.42 (0.49)	1.30 (0.55)	0.03
MHR	0.28 (0.11)	0.28 (0.11)	0.34 (0.21)	0.002
NHR	2.42 (1.14)	2.40 (1.03)	3.37 (3.36)	0.003
NHHR	2.36 (0.81)	2.35 (0.79)	2.68 (1.42)	0.02
Age	33.39 (7.04)	33.29 (7.02)	37.52 (7.10)	<0.001
BMI	22.77 (4.35)	22.76 (4.35)	23.15 (4.47)	0.36

Abbreviation: LHR, lymphocyte-to-HDL-C, ratio; MHR, monocyte-to-HDL-C, ratio; NHR, neutrophil-to-HDL-C, ratio; NHHR, non-HDL-C, to HDL-C, ratio; BMI, body mass index.

The stage of endometriosis was assessed intraoperatively according to the revised American Society for Reproductive Medicine (ASRM) staging system. Patients without definitive laparoscopic staging information were excluded from stage-related analyses. Ultimately, 105 patients with complete ASRM staging data were included in the staging analysis, of whom ASRM stages I–II were classified as early-stage endometriosis (n = 29) and stages III–IV as advanced-stage endometriosis (n = 76) (Table 2).

**TABLE 2:** Baseline characteristics of study participants according to ASRM stage of endometriosis.

Variables	All n = 105	ASRM stage I–II n = 29	ASRM stage III–IV n = 76	P value
LHR	1.31 (0.56)	1.12 (0.45)	1.38 (0.58)	0.02
MHR	0.35 (0.21)	0.27 (0.13)	0.37 (0.23)	0.006
NHR	3.41 (3.49)	2.55 (1.10)	3.73 (4.00)	0.02
NHHR	2.70 (1.47)	2.310 (0.67)	2.85 (1.65)	0.02
Age	37.20 (7.04)	37.30 (7.35)	37.10 (6.97)	0.89
BMI	23.30 (4.54)	23.10 (4.84)	23.40 (4.46)	0.82
Menarche	13.00 (1.38)	13.70 (1.34)	12.80 (1.32)	0.003
Pregnancy history				0.46
No	29 (27.62%)	6 (20.69%)	23 (30.26%)	
Yes	76 (72.38%)	23 (79.31%)	53 (69.74%)	

This study was approved by the Ethics Committee of The Eighth Affiliated Hospital of Southern Medical University (KYLS20250504) and conducted in accordance with the Declaration of Helsinki.

### 2.2 Data collection and variable definition

Fasting venous blood samples were obtained in the morning during routine clinical assessment or health examination, prior to any surgical intervention, following an overnight fast of at least 8 h. Serum lipid parameters, including high-density lipoprotein cholesterol (HDL-C) and total cholesterol (TC), were measured using standardized enzymatic assays in the hospital central laboratory. Complete blood counts, including lymphocyte, neutrophil, and monocyte counts, were determined using automated hematology analyzers as part of routine laboratory testing.

Four HDL-related lipid ratios were calculated as follows (Zhao et al., 2024; Han et al., 2025): LHR was defined as lymphocyte count divided by HDL-C; MHR as monocyte count divided by HDL-C; NHR as neutrophil count divided by HDL-C; and NHHR as the ratio of non–HDL cholesterol (TC minus HDL-C) to HDL-C. HDL-C and TC were expressed in mmol/L, and blood cell counts were expressed as ×10^9^/L.

Age and body mass index (BMI) were recorded for all participants. Among patients with endometriosis, age at menarche and pregnancy history were additionally collected from medical records.

### 2.3 External population-based comparison using NHANES

An external population-based comparison was conducted using data from the National Health and Nutrition Examination Survey (NHANES) 1999–2006 cycles. Female participants aged 20–40 years were included. Individuals with missing data on endometriosis diagnosis or any of the four HDL-related lipid ratios (LHR, MHR, NHR, NHHR) were excluded. Endometriosis was defined based on self-reported physician diagnosis. The final NHANES sample included 163 women with endometriosis and 2,863 women without endometriosis.

Laboratory measurements of lipid parameters and blood cell counts in NHANES were performed using standardized, quality-controlled protocols, ensuring comparability with routine clinical laboratory testing. Because surgical staging information was unavailable in NHANES, analyses were limited to comparisons between women with and without endometriosis to assess the consistency of association direction between the surgically confirmed clinical cohort and the NHANES population.

### 2.4 Statistical analysis

All statistical analyses were performed using R software (version 4.5.1). Baseline characteristics were presented as frequencies and percentages for categorical variables, and as means ± standard deviations (SD) for continuous variables. Between-group comparisons were conducted using the χ^2^ test, independent t-test, or Mann–Whitney U test, as appropriate. For the NHANES cohort, analyses accounted for the complex survey design. Continuous variables were expressed as weighted means ± standard errors (SE), and appropriate sampling weights were applied in all analyses.

Associations between HDL-related lipid ratios (LHR, MHR, NHR, and NHHR) and endometriosis status were examined using multivariable logistic regression models, with the control group as the reference. These models were adjusted for age and BMI. Results were reported as odds ratios (ORs) with 95% confidence intervals (CIs). To further improve comparability between the endometriosis and control groups, propensity score matching (PSM) was conducted based on age and BMI using a nearest-neighbor matching algorithm. Baseline characteristics after matching were re-evaluated, and logistic regression analyses were repeated in the matched cohort to assess the consistency of associations.

Among participants with surgically confirmed endometriosis and available staging information, additional multivariable logistic regression analyses were performed to evaluate the associations between HDL-related lipid ratios and disease stage, comparing the advanced-stage group with the early-stage group as the reference. These models were adjusted for age, BMI, age at menarche, and pregnancy history. To further assess the stability of the observed associations under an alternative weighting framework, inverse probability of treatment weighting (IPTW) based on propensity scores was applied, and weighted logistic regression models were constructed using the same covariate sets as in the primary analyses. Due to skewed distributions, all lipid ratio variables were log-transformed prior to regression analyses. After log transformation, model diagnostics indicated stable regression performance, reasonable residual distributions based on visual inspection of residual plots, and no evidence of substantial multicollinearity as assessed by variance inflation factors.

Receiver operating characteristic (ROC) curve analyses were conducted to evaluate the discriminative performance of HDL-related lipid ratios for (1) endometriosis status (endometriosis vs. control) and (2) ASRM stage classification (advanced-stage vs. early-stage endometriosis). Areas under the curve (AUCs) were calculated based on multivariable logistic regression models adjusted for relevant covariates.

All statistical tests were two-sided, and a P-value <0.05 was considered statistically significant.

## 3 Results

### 3.1 Baseline characteristics and distribution of HDL-Related lipid ratios by endometriosis status

Baseline characteristics of the study participants according to endometriosis status are summarized in Table 1. A total of 5,161 women were included, comprising 113 patients with endometriosis and 5,048 normal controls. Compared with the control group, patients with endometriosis were significantly younger (P < 0.001), whereas no significant difference in BMI was observed between the two groups (P = 0.36).

**FIGURE 1:**
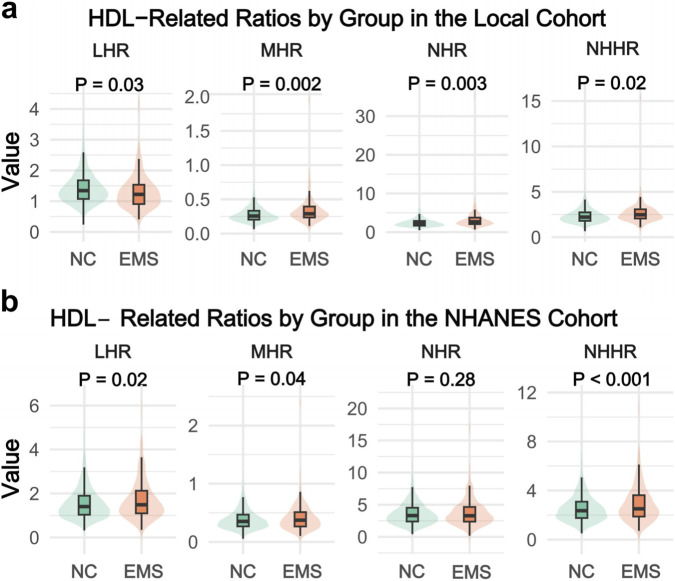
Violin plots showing the distribution of HDL-related lipid ratios between women with endometriosis (EMS) and normal controls (NC). **(a)** Local hospital-based cohort (NC: n = 5,048; EMS: n = 113). **(b)** NHANES cohort (NC: n = 2,863; EMS: n = 163). Central boxes indicate medians and interquartile ranges, with kernel density representing the distribution of values. LHR = lymphocyte-to-HDL ratio; MHR = monocyte-to-HDL ratio; NHR = neutrophil-to-HDL ratio; NHHR = non-HDL-to-HDL ratio.

In the local cohort, all four HDL-related lipid ratios differed significantly between the endometriosis (EMS) and normal control (NC) groups (Figure 1a). Specifically, levels of MHR, NHR, and NHHR were significantly higher in the EMS group than in the NC group (P = 0.002, 0.003, and 0.02, respectively), whereas LHR was significantly lower in patients with endometriosis (P = 0.03).

To further assess the consistency of these findings at the population level, group comparisons were performed in the NHANES cohort (Figure 1b). In this external population, LHR, MHR, and NHHR showed significant differences between the EMS and NC groups (P = 0.02, 0.04, and <0.001, respectively), whereas no statistically significant difference was observed for NHR (P = 0.28).

### 3.2 Associations between HDL-Related lipid ratios and endometriosis status

**FIGURE 2:**
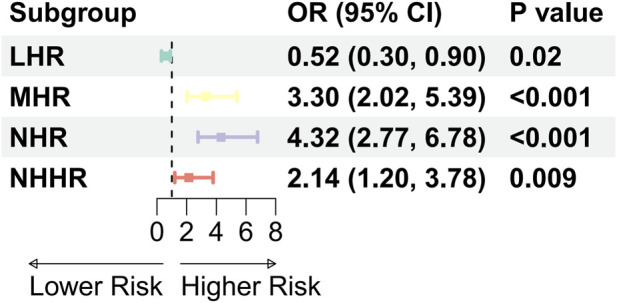
Forest plot illustrating the associations between HDL-related lipid ratios and endometriosis status in the local cohort (NC: n = 5,048; EMS: n = 113). Odds ratios (ORs) and 95% confidence intervals (CIs) were estimated using multivariable logistic regression models adjusted for age and body mass index (BMI). The normal control group served as the reference category. All lipid ratio variables were log-transformed.

Multivariable logistic regression analyses were performed to evaluate the associations between HDL-related lipid ratios and endometriosis status. After adjustment for age and BMI, LHR was inversely associated with endometriosis status (OR = 0.52, 95% CI: 0.30–0.90, P = 0.02). In contrast, higher levels of MHR (OR = 3.30, 95% CI: 2.02–5.39, P < 0.001), NHR (OR = 4.32, 95% CI: 2.77–6.78, P < 0.001), and NHHR (OR = 2.14, 95% CI: 1.20–3.78, P = 0.009) were all significantly associated with endometriosis status (Figure 2).

To further assess the robustness of these associations, PSM based on age and BMI was conducted to improve comparability between the EMS and NC groups. Baseline characteristics after matching are presented in Supplementary Table 1. In PSM-adjusted logistic regression models (Supplementary Table 2), the associations between HDL-related lipid ratios and endometriosis status remained consistent with the main analyses.

### 3.3 Associations of HDL-Related lipid ratios with ASRM stage of endometriosis

The distribution of HDL-related lipid ratios across ASRM stages was examined among 105 patients with endometriosis (Table 2). Compared with the early-stage group (ASRM I–II), patients in the advanced-stage group (ASRM III–IV) had significantly higher levels of LHR, MHR, NHR, and NHHR (all P < 0.05). In addition, age at menarche was significantly lower in the advanced-stage group than in the early-stage group (P = 0.003). No statistically significant differences were observed between the two groups in terms of age, BMI, or pregnancy history.

### 3.4 Associations between HDL-Related lipid ratios and ASRM stage of endometriosis

In multivariable logistic regression models, with the early-stage group as the reference category, all HDL-related lipid ratios were significantly associated with increased odds of being in the advanced-stage group (Table 3). Higher levels of LHR (OR = 3.97, 95% CI: 1.14–15.7, P = 0.04), MHR (OR = 6.60, 95% CI: 1.96–26.8, P = 0.004), NHR (OR = 4.65, 95% CI: 1.50–16.8, P = 0.01), and NHHR (OR = 8.03, 95% CI: 1.47–54.3, P = 0.02) were observed. All models were adjusted for age, BMI, age at menarche, and pregnancy history.

**TABLE 3:** Association between HDL-related lipid ratios and endometriosis status in logistic regression models.

Exposure	OR	95% CI	P value
LHR**	3.97	1.14, 15.70	0.04
MHR**	6.60	1.96, 26.80	0.004
NHR**	4.65	1.50, 16.80	0.01
NHHR**	8.03	1.47, 54.30	0.02

Adjusted for Age, BMI, menarche and pregnancy history.

**LHR, MHR, NHR, and NHHR, were log-transformed prior to analysis due to skewed distributions.

To further verify the robustness of the primary associations, IPTW was applied. After weighting, levels of LHR, MHR, NHR, and NHHR remained significantly higher in the advanced-stage group than in the early-stage group (all P < 0.05; Supplementary Table 3). IPTW-weighted logistic regression analyses further demonstrated associations between all four HDL-related lipid ratios and advanced-stage classification that were consistent with the main analyses (SupplementaryTable 4).

### 3.5 Discriminatory performance of HDL-Related lipid ratios

**FIGURE 3:**
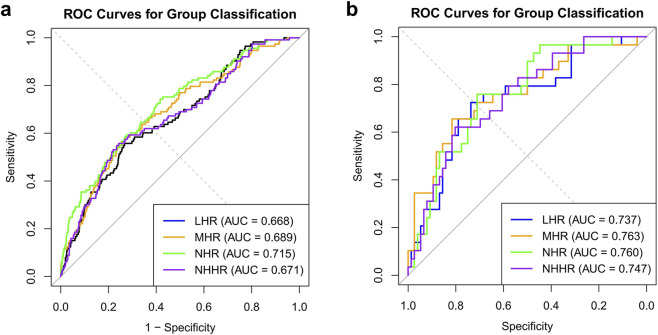
Receiver operating characteristic (ROC) curves evaluating the discriminative performance of HDL-related lipid ratios. **(a)** ROC curves for distinguishing women with endometriosis from normal controls in the local cohort, based on logistic regression models adjusted for age and BMI (NC: n = 5,048; EMS: n = 113). **(b)** ROC curves for discriminating between early-stage (ASRM stages I–II) and advanced-stage (ASRM stages III–IV) endometriosis among patients with available surgical staging data, based on models adjusted for age, BMI, age at menarche, and pregnancy history (early-stage: n = 29; advanced-stage: n = 76). The area under the curve (AUC) was calculated to quantify discriminative performance, with AUC values displayed in the legend.

ROC analyses were performed to evaluate the discriminative performance of HDL-related lipid ratios for endometriosis status and for ASRM stage classification (Figure 3). In analyses distinguishing women with endometriosis from controls, age- and BMI-adjusted ROC curves showed AUC values of 0.668 for LHR, 0.689 for MHR, 0.715 for NHR, and 0.671 for NHHR (Figure 3a). For ASRM stage classification, ROC analyses adjusted for age, BMI, age at menarche, and pregnancy history yielded AUC values of 0.737 for LHR, 0.763 for MHR, 0.760 for NHR, and 0.747 for NHHR (Figure 3b).

## 4 Discussion

This study systematically examined the associations between four HDL-related lipid ratios (LHR, MHR, NHR, and NHHR) and both endometriosis status and ASRM stage of endometriosis. The results showed that LHR was inversely associated with endometriosis status, whereas MHR, NHR, and NHHR were positively associated with endometriosis status. In addition, all four HDL-related lipid ratios were positively associated with advanced ASRM stage relative to early-stage disease. These associations remained directionally consistent in analyses using propensity score matching and inverse probability of treatment weighting, supporting the robustness of the findings. These results suggest that HDL-related lipid ratios reflect systemic metabolic–inflammatory status and may be associated with phenotypic differences across ASRM stages, supporting their potential role as adjunctive indicators for clinical stratification.

LHR, derived from lymphocyte counts and HDL levels, may reflect a chronic low-grade inflammatory state induced by increased lymphocyte activity and diminished HDL function. In this study, LHR was decreased in patients with endometriosis, in contrast to the direction observed in the NHANES population-based analysis, suggesting population-specific differences in its behavior. This discrepancy may partly reflect population heterogeneity in immune profiles, as lymphocyte subset distributions and ancestry-related immune phenotypes differ across racial groups (Slight-Webb et al., 2023; Choong et al., 1995; Wong et al., 2013). Previous studies have reported that lower LHR is associated with worse pulmonary outcomes in Chinese patients with chronic obstructive pulmonary disease (Huang et al., 2020), whereas studies in American and Iranian populations have more often used LHR as a marker of systemic inflammation in metabolic syndrome (Kolahi Ahari et al., 2024; Hashemi et al., 2024). Additionally, data from the MIMIC database have linked reduced LHR to increased mortality in patients with sepsis (Liu et al., 2023). These findings indicate that although LHR is significantly associated with endometriosis status, its interpretability as a uniform inflammation-sensitive marker may be limited across populations. Notably, in the present cohort, LHR showed a significant association with ASRM stage but not a consistent association with disease status.

MHR, based on the monocyte-to-HDL ratio, is a widely used marker for evaluating inflammation along the monocyte-lipoprotein axis. In this study, MHR was significantly elevated in patients with advanced-stage disease and positively associated with endometriosis status—findings showing a consistent direction with NHANES-based population data, suggesting cross-population consistency for endometriosis status. Although the direct role of monocytes in lesion formation remains unclear, some monocyte-derived macrophages may participate in lesion clearance (Vallvé-Juanico et al., 2022; Hogg et al., 2021). However, monocyte elevation generally reflects heightened immune activation and systemic inflammation. Moreover, inflammatory lesions may actively recruit monocytes, contributing to disease progression (Shi and Pamer, 2011; Wang et al., 2025; Hogg et al., 2020).

NHR, reflecting the balance between neutrophil-related inflammatory activity and HDL levels, has been proposed as an indicator of systemic inflammatory status. In this study, NHR showed a clear association with ASRM stage, whereas its association with endometriosis status varied across the local cohort and the NHANES population, suggesting that NHR may be more informative for ASRM stage–based stratification than for risk discrimination across different populations. This finding aligns with previous research showing that ectopic endometrial tissue is more likely to recruit neutrophils compared to eutopic endometrium (Wang et al., 2023), and that elevated levels of VEGF and IL-8 in ovarian endometriotic cyst fluid enhance neutrophil infiltration and local angiogenesis, facilitating adhesion and implantation of ectopic cells (Fasciani et al., 2000). Furthermore, neutrophil counts have been positively correlated with ASRM stage in ovarian endometriosis (Xu et al., 2020; Sabra et al., 2024). Under normal physiological conditions, HDL mitigates inflammation by suppressing the activation of both monocytes and neutrophils (Raupachova et al., 2019; Murphy et al., 2008; Murphy et al., 2011). Therefore, elevations in MHR and NHR may reflect increased chronic and acute inflammatory burdens, respectively, consistent with the systemic inflammatory milieu often observed in endometriosis.

NHHR, which integrates non-HDL cholesterol and HDL levels, reflects a combined profile of atherogenic lipoprotein burden and impaired anti-inflammatory HDL function. This ratio has been widely associated with chronic metabolic inflammation in prior studies (Wu and Gong, 2024; Zhu et al., 2015). Given that endometriosis is characterized by persistent inflammation, the pathophysiologic features captured by NHHR align closely with the disease’s underlying biology (Bulun et al., 2019). Our findings demonstrated a significant positive association between NHHR and endometriosis status, consistent with existing literature (Jiang et al., 2025). Notably, we also identified a novel association between NHHR and ASRM stage, expanding its potential clinical utility in the context of endometriosis.

Further ROC analyses indicated that all four HDL-related lipid ratios showed better discriminatory performance for ASRM stage classification than for distinguishing endometriosis cases from controls, suggesting greater relevance for ASRM stage–based stratification. For ASRM stage classification, MHR yielded the highest AUC (0.763), followed by NHR (0.760), NHHR (0.747), and LHR (0.737). When distinguishing endometriosis cases from controls, NHR also demonstrated comparatively stronger discrimination (AUC = 0.715) than the other ratios. Collectively, these findings suggest that HDL-related lipid ratios may have potential value as adjunctive indicators for clinical stratification, with MHR showing the strongest discriminatory ability for ASRM stage classification and NHR exhibiting comparatively stronger performance in distinguishing endometriosis status.

The strengths of this study include the use of surgically confirmed clinical data, ensuring accurate diagnosis and staging, as well as the robustness of the observed associations across different analytical frameworks. To our knowledge, this is the first study to systematically compare multiple HDL-related lipid ratios in relation to both endometriosis status and ASRM stage, highlighting their potential value as adjunctive blood-based indicators for clinical assessment. However, several limitations must be acknowledged. First, in NHANES, endometriosis was defined by self-reported physician diagnosis; therefore, these analyses are limited to cautious interpretation of status-level associations and do not support ASRM stage–related conclusions. Second, the cross-sectional design precludes causal inference. Third, although our findings provide supportive evidence for potential associations between HDL-related lipid ratios and ASRM stage, the number of early-stage cases in the clinical cohort was relatively limited, resulting in wide confidence intervals for some ASRM stage–related estimates and reduced statistical precision. Accordingly, these findings may represent preliminary exploratory evidence and warrant further validation in larger, prospective cohorts. In addition, due to the complex etiology of endometriosis, some residual confounding may persist despite adjustment. Nonetheless, from a clinical perspective, this study provides the first comprehensive evaluation of LHR, MHR, NHR, and NHHR in both endometriosis status identification and ASRM stage classification, highlighting their potential role as adjunctive indicators for clinical stratification and management. Future research should adopt prospective, multicenter cohort designs and incorporate mechanistic investigations to further elucidate the roles of HDL-related lipid ratios in the pathogenesis and progression of endometriosis.

## Data Availability

The clinical dataset from the Chinese hospital cohort is not publicly available due to privacy concerns but is available from the corresponding author upon reasonable request. The NHANES data are publicly available from the official website of the National Health and Nutrition Examination Survey (https://www.cdc.gov/nchs/nhanes/).

## References

[B1] Allaire C. Bedaiwy M. A. Yong P. J. (2023). Diagnosis and management of endometriosis. CMAJ 195, E363–E371. 10.1503/cmaj.220637 36918177 PMC10120420

[B2] As-Sanie S. Mackenzie S. C. Morrison L. Schrepf A. Zondervan K. T. Horne A. W. (2025). Endometriosis: a review. JAMA 334, 64–78. 10.1001/jama.2025.2975 40323608 PMC13498397

[B3] Bulun S. E. Yilmaz B. D. Sison C. Miyazaki K. Bernardi L. Liu S. (2019). Endometriosis. Endocr. Rev. 40, 1048–1079. 10.1210/er.2018-00242 30994890 PMC6693056

[B4] Chen Z. Li R. Guo J. Ye X. Zhou Y. Cao M. (2025). Association between remnant cholesterol (RC) and endometriosis: a cross-sectional study based on NHANES data. Lipids Health Dis. 24, 2. 10.1186/s12944-024-02422-4 39754185 PMC11699680

[B5] Chiesa S. T. Charakida M. McLoughlin E. Nguyen H. C. Georgiopoulos G. Motran L. (2019). Elevated high-density lipoprotein in adolescents with type 1 diabetes is associated with endothelial dysfunction in the presence of systemic inflammation. Eur. Heart J. 40, 3559–3566. 10.1093/eurheartj/ehz114 30863865 PMC6855140

[B6] Choong M. L. Ton S. H. Cheong S. K. (1995). Influence of race, age and sex on the lymphocyte subsets in peripheral blood of healthy Malaysian adults. Ann. Clin. Biochem. 32, 532–539. 10.1177/000456329503200603 8579284

[B7] Erdoğan K. Sanlier N. T. Özen E. U. Erol S. Kahyaoğlu I. Neselioglu S. (2023). Evaluation of dysfunctional HDL by myeloperoxidase/paraoxonase ratio in unexplained infertility patients undergoing IVF/ICSI. J. Clin. Med. 12, 1506. 10.3390/jcm12041506 36836040 PMC9964667

[B8] Fasciani A. D’Ambrogio G. Bocci G. Monti M. Genazzani A. R. Artini P. G. (2000). High concentrations of the vascular endothelial growth factor and interleukin-8 in ovarian endometriomata. Mol. Hum. Reprod. 6, 50–54. 10.1093/molehr/6.1.50 10611260

[B9] Han Y. Gao Y. Qiu M. Wang Y. Li S. Guo M. (2025). Association between non-high-density lipoprotein cholesterol to high-density lipoprotein cholesterol ratio and cerebral atherosclerotic stenosis: a retrospective study. Lipids Health Dis. 24, 145. 10.1186/s12944-025-02555-0 40241203 PMC12004609

[B10] Hashemi S. M. Kheirandish M. Rafati S. Ghazalgoo A. Amini-Salehi E. Keivanlou M.-H. (2024). The association between neutrophil and lymphocyte to high-density lipoprotein cholesterol ratio and metabolic syndrome among Iranian population, finding from bandare kong cohort study. Lipids Health Dis. 23, 393. 10.1186/s12944-024-02378-5 39604922 PMC11603836

[B11] Higashi Y. (2023). Endothelial function in dyslipidemia: roles of LDL-Cholesterol, HDL-cholesterol and triglycerides. Cells 12, 1293. 10.3390/cells12091293 37174693 PMC10177132

[B12] Hogg C. Horne A. W. Greaves E. (2020). Endometriosis-associated macrophages: origin, phenotype, and function. Front. Endocrinol. 11, 11. 10.3389/fendo.2020.00007 32038499 PMC6989423

[B13] Hogg C. Panir K. Dhami P. Rosser M. Mack M. Soong D. (2021). Macrophages inhibit and enhance endometriosis depending on their origin. Proc. Natl. Acad. Sci. U. S. A. 118, e2013776118. 10.1073/pnas.2013776118 33536334 PMC8017702

[B14] Huang Y. Jiang B. Miao X. Ma J. Wang J. Ding K. (2020). The relationship of lymphocyte to high-density lipoprotein ratio with pulmonary function in COPD. Int. J. Chron. Obstruct Pulmon Dis. 15, 3159–3169. 10.2147/COPD.S276372 33293805 PMC7718883

[B15] Jiang P. Zhang X. Huang H. Sun Z. Hu W. Li Y. (2025). Study on the relationship between the non-HDL/HDL cholesterol ratio (NHHR) and endometriosis: a cross-sectional analysis utilizing the NHANES dataset. Lipids Health Dis. 24, 179. 10.1186/s12944-025-02590-x 40375237 PMC12082855

[B16] Kolahi Ahari R. Akbari N. Babaeepoor N. Fallahi Z. Saffar Soflaei S. Ferns G. (2024). Association of three novel inflammatory markers: lymphocyte to HDL‐C ratio, high‐sensitivity c‐reactive protein to HDL‐C ratio and high‐sensitivity c‐reactive protein to lymphocyte ratio with metabolic syndrome. Endocrinol. Diabetes Metab. 7, e00479. 10.1002/edm2.479 38590230 PMC11002532

[B17] Kvaskoff M. Mu F. Terry K. L. Harris H. R. Poole E. M. Farland L. (2015). Endometriosis: a high-risk population for major chronic diseases? Hum. Reprod. Update 21, 500–516. 10.1093/humupd/dmv013 25765863 PMC4463000

[B18] Leone Roberti Maggiore U. Chiappa V. Ceccaroni M. Roviglione G. Savelli L. Ferrero S. (2024). Epidemiology of infertility in women with endometriosis. Best Pract. & Res. Clin. Obstetrics & Gynaecol. 92, 102454. 10.1016/j.bpobgyn.2023.102454 38183767

[B19] Li Y. Guo X. Ge J. Li Q. Chen X. Zhu Y. (2025). Sex differences in associations of metabolic inflammation and insulin resistance with incident type 2 diabetes mellitus: a retrospective cohort of adults with annual health examinations. Lipids Health Dis. 24, 50. 10.1186/s12944-025-02473-1 39953587 PMC11829553

[B20] Liang J. Xie Y. Li P. Li H. Li P. Huang Z. (2025). The non-high-density lipoprotein cholesterol to high-density lipoprotein cholesterol ratio and its combination with obesity indicators as a predictor of all cause and cardiovascular mortality in non-diabetic individuals. BMC Public Health 25, 1513. 10.1186/s12889-025-22789-y 40269817 PMC12016409

[B21] Liu W. Tao Q. Xiao J. Du Y. Pan T. Wang Y. (2023). Low lymphocyte to high-density lipoprotein ratio predicts mortality in sepsis patients. Front. Immunol. 14, 1279291. 10.3389/fimmu.2023.1279291 37901205 PMC10601636

[B22] Ma Y. Wu F. Yu Z. Yang L. (2025). Evaluating the association between lipidome and female reproductive diseases through comprehensive Mendelian randomization analyses. Sci. Rep. 15, 2448. 10.1038/s41598-025-86794-2 39828767 PMC11743779

[B23] Melo A. S. Rosa-e-Silva J. C. Rosa-e-Silva A. C. J. de S. Poli-Neto O. B. Ferriani R. A. Vieira C. S. (2009). Unfavorable lipid profile in women with endometriosis. Fertil. Sterility 93, 2433–2436. 10.1016/j.fertnstert.2009.08.043 19969295

[B24] Murphy A. J. Woollard K. J. Hoang A. Mukhamedova N. Stirzaker R. A. McCormick S. P. A. (2008). High-density lipoprotein reduces the human monocyte inflammatory response. Arteriosclerosis, Thrombosis, Vasc. Biol. 28, 2071–2077. 10.1161/ATVBAHA.108.168690 18617650

[B25] Murphy A. J. Woollard K. J. Suhartoyo A. Stirzaker R. A. Shaw J. Sviridov D. (2011). Neutrophil activation is attenuated by high-density lipoprotein and apolipoprotein A-I in *in vitro* and *in vivo* models of inflammation. Arteriosclerosis, Thrombosis, Vasc. Biol. 31, 1333–1341. 10.1161/ATVBAHA.111.226258 21474825

[B26] Peng D. Zhong W. Wang Y. Fu Y. Shang W. (2024). The relationship between blood lipids and endometriosis: a cross-sectional study from NHANES (1999–2006) and a bidirectional Mendelian randomization study. J. Psychosomatic Obstetrics Gynaecology 45, 2441196. 10.1080/0167482X.2024.2441196 39703074

[B27] Raupachova J. Kopecky C. Cohen G. (2019). High-density lipoprotein from chronic kidney disease patients modulates polymorphonuclear leukocytes. Toxins (Basel) 11, 73. 10.3390/toxins11020073 30717079 PMC6409858

[B28] Rysz J. Gluba-Brzózka A. Rysz-Górzyńska M. Franczyk B. (2020). The role and function of HDL in patients with chronic kidney disease and the risk of cardiovascular disease. Int. J. Mol. Sci. 21, 601. 10.3390/ijms21020601 31963445 PMC7014265

[B29] Sabra ASIM Moselhy S. N. A. Eldin AKMZ (2024). Systemic inflammatory indices as a non-invasive grading modality for endometriosis: a comparative study *versus* exploratory laparoscopy. Rev. Bras. Ginecol. Obstet. 46, e-rbgo84. 10.61622/rbgo/2024rbgo84 39669306 PMC11637453

[B30] Saei Ghare Naz M. Noroozzadeh M. Ardebili S. N. Mousavi M. Azizi F. Ramezani Tehrani F. (2024). Cardio‐metabolic risk profile of women with endometriosis: a population‐based study. Endocrinol. Diabetes Metab. 7, e70008. 10.1002/edm2.70008 39400459 PMC11471882

[B31] Saunders P. T. K. Horne A. W. (2025). Endometriosis: new insights and opportunities for relief of symptoms. Biol. Reprod. 113, 1029–1043. 10.1093/biolre/ioaf164 40704733 PMC12621312

[B32] Shi C. Pamer E. G. (2011). Monocyte recruitment during infection and inflammation. Nat. Rev. Immunol. 11, 762–774. 10.1038/nri3070 21984070 PMC3947780

[B33] Slight-Webb S. Thomas K. Smith M. Wagner C. A. Macwana S. Bylinska A. (2023). Ancestry-based differences in the immune phenotype are associated with lupus activity. JCI Insight 8, e169584. 10.1172/jci.insight.169584 37606045 PMC10543734

[B34] Sun H. Liu H. Li J. Kou J. Yang C. (2024). Analysis of the clinical predictive value of the novel inflammatory indices SII, SIRI, MHR and NHR in patients with acute myocardial infarction and their extent of coronary artery disease. J. Inflamm. Res. 17, 7325–7338. 10.2147/JIR.S479253 39429847 PMC11490208

[B35] Terry K. L. Shafrir A. Laliberte A. Vitonis A. F. Garbutt K. DePari M. (2025). Circulating inflammatory biomarkers and endometriosis lesion characteristics in the WisE consortium. NPJ Womens Health 3, 62. 10.1038/s44294-025-00110-x 41180139 PMC12578628

[B36] Vallvé-Juanico J. George A. F. Sen S. Thomas R. Shin M.-G. Kushnoor D. (2022). Deep immunophenotyping reveals endometriosis is marked by dysregulation of the mononuclear phagocytic system in endometrium and peripheral blood. BMC Med. 20, 158. 10.1186/s12916-022-02359-4 35421980 PMC9011995

[B37] Van Gestel H. Bafort C. Meuleman C. Tomassetti C. Vanhie A. (2024). The prevalence of endometriosis in unexplained infertility: a systematic review. Reprod. Biomed. Online 49, 103848. 10.1016/j.rbmo.2024.103848 38943813

[B38] Wang X. Jia Y. Li D. Guo X. Zhou Z. Qi M. (2023). The abundance and function of neutrophils in the endometriosis systemic and pelvic microenvironment. Mediat. Inflamm. 2023, 1481489. 10.1155/2023/1481489 36762287 PMC9904898

[B39] Wang Z. Zhan C. Liao L. Luo Y. Lin S. Yan S. (2024). Bidirectional causality between the levels of blood lipids and endometriosis: a two-sample Mendelian randomization study. BMC Womens Health 24, 387. 10.1186/s12905-024-03213-w 38965508 PMC11223312

[B40] Wang X. Wu N. Xue Q. (2025). Macrophages in endometriosis: key roles and emerging therapeutic Opportunities—a narrative review. Reprod. Biol. Endocrinol. 23, 134. 10.1186/s12958-025-01471-3 41121289 PMC12538831

[B41] Wong W. S. Lo A. W. I. Siu L. P. Leung J. N. S. Tu S. P. Tai S. W. (2013). Reference ranges for lymphocyte subsets among healthy Hong Kong Chinese adults by single-platform flow cytometry. Clin. Vaccine Immunol. 20, 602–606. 10.1128/CVI.00476-12 23408529 PMC3623424

[B42] Wu R. Gong H. (2024). The association between non-high-density lipoprotein cholesterol to high-density lipoprotein cholesterol ratio and chronic obstructive pulmonary disease: the mediating role of dietary inflammatory index. Front. Nutr. 11, 1427586. 10.3389/fnut.2024.1427586 39315013 PMC11416962

[B43] Xiong H. Yu Z. (2025). Association between systemic inflammation indicators and psoriasis: a cross-sectional study from NHANES. Front. Immunol. 16, 1556487. 10.3389/fimmu.2025.1556487 40181962 PMC11965919

[B44] Xu H. Zhao J. Lu J. Sun X. (2020). Ovarian endometrioma infiltrating neutrophils orchestrate immunosuppressive microenvironment. J. Ovarian Res. 13, 44. 10.1186/s13048-020-00642-7 32334621 PMC7183111

[B45] Yu X.-H. Zhang D.-W. Zheng X.-L. Tang C.-K. (2019). Cholesterol transport system: an integrated cholesterol transport model involved in atherosclerosis. Prog. Lipid Res. 73, 65–91. 10.1016/j.plipres.2018.12.002 30528667

[B46] Zhao J. Zheng Q. Ying Y. Luo S. Liu N. Wang L. (2024). Association between high-density lipoprotein-related inflammation index and periodontitis: insights from NHANES 2009–2014. Lipids Health Dis. 23, 321. 10.1186/s12944-024-02312-9 39342327 PMC11439298

[B47] Zhu L. Lu Z. Zhu L. Ouyang X. Yang Y. He W. (2015). Lipoprotein ratios are better than conventional lipid parameters in predicting coronary heart disease in Chinese Han people. Pol. Heart J. (Kardiologia Polska) 73, 931–938. 10.5603/KP.a2015.0086 25985729

[B48] Zolbin M. M. Mamillapalli R. Nematian S. E. Goetz L. Taylor H. S. (2019). Adipocyte alterations in endometriosis: reduced numbers of stem cells and microRNA induced alterations in adipocyte metabolic gene expression. Reprod. Biol. Endocrinol. 17, 36. 10.1186/s12958-019-0480-0 30982470 PMC6463663

