# Supplementary material for: HDL-related lipid ratios reflecting metabolic inflammation are associated with endometriosis status and ASRM stage
Source: Front Physiol. 2026 Feb 20;17:1723135. doi: 10.3389/fphys.2026.1723135 (PMC12963222; doi:10.3389/fphys.2026.1723135)
Supplement: Supplementary file 1 [file Table1.docx]

**Supplementary Table 1.** Baseline Characteristics of Study Participants According to Endometriosis Status based on PSM

| **Variables** | **All**  **n=5,161** | **Control**  **n=5,048** | **Endometriosis**  **n=113** | **P value** |
| --- | --- | --- | --- | --- |
| **LHR** | 1.39 (0.53) | 1.41 (0.52) | 1.31 (0.55) | 0.09 |
| **MHR** | 0.29 (0.14) | 0.28 (0.11) | 0.34 (0.21) | **0.002** |
| **NHR** | 2.62 (1.94) | 2.37 (0.98) | 3.37 (3.38) | **0.002** |
| **NHHR** | 2.53 (1.06) | 2.47 (0.91) | 2.69 (1.42) | 0.13 |
| **Age** | 37.45 (7.23) | 37.46 (7.31) | 37.41 (7.03) | 0.95 |
| **BMI** | 22.91 (4.26) | 22.86 (4.23) | 23.06 (4.36) | 0.68 |
| Abbreviation: LHR = lymphocyte-to-HDL-C ratio; MHR = monocyte-to-HDL-C ratio; NHR = neutrophil-to-HDL-C ratio; NHHR = non-HDL-C to HDL-C ratio; BMI = body mass index. | | | | |

**Supplementary Table 2.** Association between HDL-related lipid ratios and endometriosis status in logistic regression models based on PSM

| **Exposure** | **OR** | **95% CI** | **P value** |
| --- | --- | --- | --- |
| **LHR **** | 0.48 | 0.26, 0.89 | **0.02** |
| **MHR **** | 3.14 | 1.79, 5.63 | **< 0.001** |
| **NHR **** | 4.15 | 2.45, 7.28 | **< 0.001** |
| **NHHR **** | 1.96 | 1.03, 3.79 | **0.04** |
| Abbreviation: CI = Confidence Interval.  Adjusted for Age, BMI, Menarche and Pregnancy History.  ****** LHR, MHR, NHR and NHHR were log-transformed. | | | |

**Supplementary Table 3.** IPTW-Adjusted Baseline Characteristics of Study Participants by Endometriosis Stage.

| **Variables** | **All**  **n=445** | **ASRM Stage I–II**  **n=48** | **ASRM Stage III–IV**  **n=397** | **P value** |
| --- | --- | --- | --- | --- |
| **LHR** | 1.30 (0.55) | 1.10 (0.44) | 1.33 (0.56) | **0.04** |
| **MHR** | 0.36 (0.22) | 0.27 (0.12) | 0.37 (0.22) | **0.001** |
| **NHR** | 3.62 (3.70) | 2.60 (1.18) | 3.75 (3.89) | **0.03** |
| **NHHR** | 2.83 (1.53) | 2.35 (0.71) | 2.89 (1.60) | **0.03** |
| **Age** | 37.14 (7.07) | 37.63 (7.36) | 37.08 (7.06) | 0.50 |
| **BMI** | 23.73 (4.66) | 23.48 (5.25) | 23.76 (4.60) | 0.60 |
| **Menarche (years)** | 12.37 (1.46) | 14.08 (1.50) | 12.16 (1.31) | **<0.001** |
| **Pregnancy History** |  |  |  | 0.06 |
| **No** | 149 (33.48%) | 8 (16.67%) | 141 (35.52%) |  |
| **Yes** | 296 (66.52%) | 40 (83.33%) | 256 (64.48%) |  |
| Abbreviation: IPTW = inverse probability of treatment weighting; LHR = lymphocyte-to-HDL-C ratio; MHR = monocyte-to-HDL-C ratio; NHR = neutrophil-to-HDL-C ratio; NHHR = non-HDL-C to HDL-C ratio; BMI = body mass index. | | | | |

**Supplementary Table 4.** Association Between HDL-Related Lipid Ratios and advanced-stage (ASRM III–IV vs I–II) endometriosis in IPTW-Weighted Logistic Regression Models.

| **Exposure** | **OR** | **95% CI** | **P value** |
| --- | --- | --- | --- |
| **LHR **** | 4.08 | 1.25, 13.30 | **0.02** |
| **MHR **** | 7.76 | 1.98, 30.40 | **0.004** |
| **NHR **** | 4.97 | 1.63, 15.10 | **0.005** |
| **NHHR**** | 8.94 | 1.57, 50.80 | **0.01** |
| Abbreviation: CI = Confidence Interval.  Adjusted for Age, BMI, Menarche and Pregnancy History.  ****** LHR, MHR, NHR and NHHR were log-transformed. | | | |
